# Hydrothermal Synthesis of a Cellular NiO Film on Carbon Paper as a Promising Way to Obtain a Hierarchically Organized Electrode for a Flexible Supercapacitor

**DOI:** 10.3390/ma16155208

**Published:** 2023-07-25

**Authors:** Tatiana L. Simonenko, Nikolay P. Simonenko, Philipp Yu. Gorobtsov, Elizaveta P. Simonenko, Nikolay T. Kuznetsov

**Affiliations:** Kurnakov Institute of General and Inorganic Chemistry of the Russian Academy of Sciences, 31 Leninsky pr., Moscow 119991, Russia; egorova.offver@gmail.com (T.L.S.); n_simonenko@mail.ru (N.P.S.); phigoros@gmail.com (P.Y.G.); ntkuz@igic.ras.ru (N.T.K.)

**Keywords:** hydrothermal synthesis, thin films, hierarchical microstructure, nanosheets, NiO, flexible electrode, supercapacitor

## Abstract

The formation of a cellular hierarchically organized NiO film on a carbon paper substrate under hydrothermal conditions using triethanolamine as a base has been studied. The thermal behavior of the carbon paper substrate with the applied semi-product shell was studied using synchronous thermal analysis (TGA/DSC) and it was demonstrated that such modification of the material surface leads to a noticeable increase in its thermal stability. Using scanning electron microscopy (SEM), it was shown that the NiO film grown on the carbon fiber surface is characterized by a complex cellular morphology, organized by partially layered individual nanosheets of about 4–5 nm thickness and lateral dimensions up to 1–2 μm, some edges and folds of which are located vertically relative to the carbon fiber surface. The surface of the obtained material was also examined using atomic force microscopy (AFM), and the electronic work function of the oxide shell surface was evaluated using the Kelvin probe force microscopy (KPFM) method. The electrochemical parameters of the obtained flexible NiO/CP electrode were analyzed: the dependence of the specific capacitance on the current density was determined and the stability of the material during cycling was studied, which showed that the proposed approach is promising for manufacturing hierarchically organized electrodes for flexible supercapacitors.

## 1. Introduction

Currently, a transition to a low-carbon economy and the development of distributed energy require not only the creation of alternative power generation systems [1,2], but also storage devices [3,4,5]. Among the various currently available energy storage technologies, supercapacitors are especially in demand and ensure the operation of systems requiring high power, charging/discharging rates, and cyclic stability, and they possess long life and a wide operating temperature range (from −40 to 70 °C). In particular, supercapacitors appear very promising in the field of electric transport, memory and uninterruptible power supply systems, in the creation of modern renewable energy production plants both as an independent element and in combination with batteries, and also find wide application in the field of portable electronics [4,6,7,8,9].

It can be seen that the direction of modern microelectronics evolution is increasingly shifting from rigid to flexible printed circuits, functional and structural elements, as well as their miniaturization in order to create flexible and wearable devices [10,11,12]. This calls for innovation and adaptation of existing materials and technologies to develop flexible and stretchable planar supercapacitors while maintaining their mechanical strength and high performance characteristics, which in turn depend on the active electrode material, as well as the supporting substrate. Some of the most popular substrate materials for such devices today are different carbon materials (e.g., carbon fiber, carbon paper, carbon veil), whose advantages include high electrical conductivity, mechanical strength with a large number of bending/folding cycles, environmental friendliness, and high availability of these materials [13,14]. Transition metal oxides (TMO) can be highlighted as the active electrode material of supercapacitors, possessing a pseudocapacitance effect, which leads to higher specific capacitance values compared to electric double-layer capacitors (EDLC) [15,16,17,18]. For example, nickel oxide is one of the most popular electrode materials owing to its high theoretical specific capacitance (3750 F/g), chemical stability, low toxicity, and wide availability [10,19,20]. Nevertheless, there are a several factors that limit the achievement of high capacitance values for transition metal oxides in practice. First of all, it concerns insufficiently high electrical conductivity, leading to increased sheet resistance and charge transfer resistance, which ultimately manifests itself in increased equivalent series resistance (R_ESR_) of the device in general [21]. A promising approach to the above problem is the formation of hybrid supercapacitor electrodes that include both materials with a pseudocapacitive effect (for example, TMO) and carbon materials operating as EDLC materials, which makes it possible to achieve high values of both ionic and electronic conductivity due to the synergistic effect of different natural components [14,22,23].

When creating supercapacitor materials, increased attention is paid to the specific features of their microstructure in order to ensure high dispersity and increased specific surface area, which increases the number active sites capable of participating in electrochemical reactions that occur during the electrode’s operation. In this context, as a rule, the most popular methods for the directed synthesis of materials for alternative energy devices with the desired characteristics are liquid-phase methods, which enable one to vary a large number of synthesis parameters and influence the functional properties of the resulting materials [24,25,26,27,28,29,30,31,32]. Among this group of methods, hydrothermal synthesis is particularly noteworthy; depending on the process conditions (temperature, reactor pressure, synthesis duration, concentration and type of reagents used), it allows the formation of both nanopowders and coatings with different microstructure (particle size and shape, as well as pores), crystallinity degree, and type and degree of crystal structure defects [33,34,35,36,37]. In addition, the hydrothermal method allows the formation of anisotropic (including cellular and hierarchically organized) materials, which, usually, improve the functional characteristics of devices on their basis [38,39,40,41,42].

The hydrothermal synthesis of nickel oxide or nickel hydroxide involves popular bases (or their combinations) such as urea, sodium hydroxide, and ammonia hydrate [43,44,45,46]. Additional use of structure-forming agents (e.g., ammonium or sodium oxalates [47,48,49,50,51]) and surfactants (ethylene glycol, polyethylene glycol [48,49]) is also possible. We have previously investigated the peculiarities of the hydrothermal synthesis of nickel oxide as nanopowder with triethanolamine, which serves not only as a base but also as a complexing and structure-forming agent [52]. Proceeding from the results obtained [52], we selected the hydrothermal synthesis conditions for nickel oxide in the form of a film. The present work is a logical development of the previous one and is aimed at the formation of a flexible electrode based on a cellular hierarchically organized NiO film grown on a carbon paper (CP) substrate; further, it is aimed at studying the microstructure features of the resulting composite and its functional characteristics to evaluate the prospects of the proposed material as an electrode for modern flexible supercapacitors.

## 2. Materials and Methods

### 2.1. Hydrothermal Growth of NiO Film

The formation of a nickel oxide film on a carbon paper surface was performed according to the following procedure: At the first stage, a reaction system of 8.5 mL was prepared, which was an aqueous solution of nickel chloride (c = 0.005 mol/L; NiCl_2_·6H_2_O, >98%, RUSHIM, Moscow, Russia) and triethanolamine (c = 0.010 mol/L; C_6_H_15_NO_3_, 99%, Chimmed, Moscow, Russia). The obtained system was then transferred into a 25 mL stainless steel autoclave with a Teflon liner in which a cleaned carbon paper substrate (overall dimensions: 20 × 7 × 0.1 mm) was placed beforehand. Then, the system was heat-treated at 200 °C (heating rate of 1.5 °C/min) for 1 h, resulting in the formation of a semi-product film on the carbon fiber surface. After natural cooling of the autoclave, the substrate with the film was removed, washed with distilled water, dried (50 °C, 2 h), and subjected to additional heat treatment under thermal analysis conditions (300 °C, 2 h; in an air flow) to decompose semi-product and crystallize target nickel oxide.

### 2.2. Characterization of the Obtained Electrode

The thermal behavior of the film grown by the hydrothermal method on the carbon paper surface, as well as its crystallization, were studied simultaneously under synchronous thermal analysis conditions (SDT Q-600 thermal analyzer, TA Instruments, New Castle, DE, USA) in the temperature interval of 25–300 °C at a rate of 10°/min in an air flow of 250 mL/min, with further exposure at 300 °C for 2 h.

The microstructure features of the obtained film on the CP surface were studied by scanning electron microscopy (SEM; NVision-40, Carl Zeiss, Inc., Oberkochen, Germany) using secondary electron detectors (Everhart-Thornley SE2 detector, Inlens detector) and a backscattered electron detector (ESB) at an accelerating voltage of 1 kV. Elemental analysis and mapping of elements’ distribution over the carbon fiber surface were performed in the framework of SEM using the INCA X-MAX 80 X-ray spectral elemental microanalysis system (Oxford Instruments, Abingdon, United Kingdom) at an accelerating voltage of 20 kV and a focal distance of 5 mm.

The surface of the grown NiO film was also studied using different atomic force microscopy techniques on the Solver PRO-M microscope (NT-MDT, Zelenograd, Russia). Thus, using a tapping mode, the material’s topography was studied. Simultaneously, the surface was examined in the phase contrast mode for topographic image refinement, and surface potential distribution maps were plotted using Kelvin probe force microscopy. Using these maps, the electronic work function values of the material surface were calculated. ETALON series probes with W_2_C-conductive coating (ScanSens, Bremen, Germany) having a tip radius <35 nm were used for all AFM measurements.

Electrochemical properties of the obtained sample were studied using a P-40X potentiostat–galvanostat equipped with an FRA-24M electrochemical impedance measurement module (Electrochemical Instruments, Chernogolovka, Russia) within the three-electrode scheme. Carbon paper with a cellular nickel oxide film grown on its surface was the working electrode in this case, while the Ag/AgCl electrode and graphite rod were used as reference and counter electrodes, respectively. The material was characterized by cyclic voltammetry (CVA), galvanostatic charge–discharge (GCD), and electrochemical impedance spectroscopy (EIS). An aqueous KOH solution (c = 3 mol/L) was used as the electrolyte.

## 3. Results and Discussion

**Figure 1:**
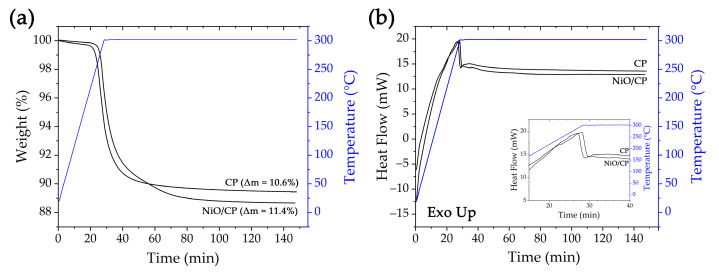
TGA (**a**) and DSC (**b**) curves of the initial carbon paper and CP after growing the semiproduct film on its surface. The temperature curve is shown in blue.

The initial carbon paper, after growing a semi-product film on its surface, was studied further by means of synchronous thermal analysis (Figure 1). This method was necessary to control the crystallization process of the NiO film on the carbon fiber surface, as well as to determine the mass fraction of active material in the resulting electrode. As can be seen from the TGA curves (Figure 1a) when heating the samples in the first stage, there is a linear weight loss—up to 200 °C for the initial carbon paper and up to 210 °C for the composite material. At the same time, for the initial CP at this stage, Δm has a value two times higher (0.30%) than in the case of the sample with modified surface (0.15%). Such differences in the material’s behavior may be related to the transformation of the material microstructural characteristics during hydrothermal treatment, which probably resulted in the removal of weakly bound sorbed molecules of atmospheric gases from the substrate surface. At the same time, modification of the CP surface with a semi-product shell followed by drying of the material prevented active sorption of atmospheric gases before the thermal analysis.

Further heating of both samples shows an intensification of the mass loss, which may be due to the oxidation and removal of the binder used in CP fabrication. As can be seen from the corresponding DSC curves (Figure 1b), for both samples, this process is accompanied by an exothermic effect. At the same time, the application of the semi-product shell to the CP surface also resulted in a shift of the exo-effect maximum, referring to the highest rate of the process at the corresponding temperature, by 10°, from 288 to 298 °C. The shift of the initiation and the active phase of this process (by 10°) for the modified CP to the region of higher temperatures may be related to the fact that the formed semi-product shell performs a protective function, slowing down the removal of the polymer binder from the carbon fiber surface. From the TGA curves, it can also be seen that the second stage of weight loss for the initial carbon paper is generally completed in 60 min from the start of heating (in 30 min from the beginning of exposure at 300 °C). For the modified sample, mass stabilization is observed after 90 min (60 min after the exposure at 300 °C begins). Furthermore, the shell application on the carbon fiber surface leads to an increase in the final Δm value (11.4%) during thermal analysis in comparison with the initial carbon paper (10.6%). Considering the differences in the magnitude of the total mass loss and the duration of the material’s mass stabilization during heat treatment, it can be assumed that in the case of the modified sample, the intermediate decomposition, accompanied by the water molecule release, makes a significant contribution to this process.

As we have shown previously [52], at the synthesis stage, under appropriate conditions (reaction system composition, reagent concentrations, and heat treatment conditions), β-Ni(OH)_2_ is formed as a semi-product containing a small amount of Ni_2_(CO_3_)(OH)_2_ as an impurity. Based on the difference between the mass loss values for the initial and modified carbon paper (about 0.8%), the mass of the NiO shell material formed as a result of heat treatment was estimated. Thus, due to the fact that the value of Δm = 0.8% corresponds to the mass of water molecules formed and evaporated during the decomposition of the semi-product at the level of 0.03 mg, the mass of the β-Ni(OH)_2_ film in this case is about 0.16 mg, and the mass of the formed nickel oxide film has a value of about 0.13 mg (given the final sample mass of about 3.54 mg, the mass fraction of the NiO shell was about 3.6%). Thus, by means of synchronous thermal analysis, the behavior of the initial and modified carbon paper was studied and the weight of the NiO film formed on the CP surface was estimated.

**Figure 2:**
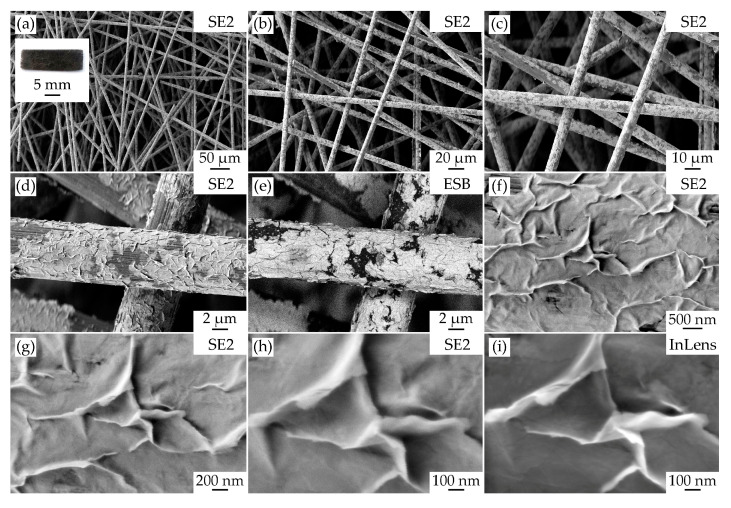
Microstructure of the obtained NiO/CP electrode surface, studied by scanning electron microscopy using different detectors (**a**–**i**), as well as the appearance of the sample ((**a**), inset).

The microstructural features of the obtained NiO/CP composite electrode were examined by scanning electron microscopy (Figure 2). As can be seen from the micrographs (Figure 2a–c), the sample consists of chaotically distributed carbon fibers with a thickness of about 7 μm. At the same time, the main surface of the fibers is covered by the hydrothermally grown oxide shell. When the surface of a single fiber is studied with the SE2 secondary electron detector, it can be observed (Figure 2d) that the NiO film is characterized by a complex morphology, and the image (Figure 2e) obtained with the ESB backscattered electron detector clearly demonstrates the distribution by mean atomic number, where the shell material with a higher molar mass value is significantly different from the carbon fiber underneath it. The results of a more detailed analysis of the oxide shell’s morphology (Figure 2f–i) indicate that the formed film has a developed cellular microstructure which, due to the large number of active sites capable of participating in electrochemical reactions, is more preferable to ensure high efficiency of supercapacitor electrodes. It can be seen that the NiO film is organized from partially overlapping individual nanosheets with a thickness of about 4–5 nm and lateral dimensions up to 1–2 μm. The mutual arrangement of the individual nanosheets, some edges and folds of which are vertical to the surface of the carbon fiber, can be clearly seen when studying the material surface with an InLens secondary electron detector (Figure 2i). The results show that the main surface of the oxide film consists of several mutually overlapping NiO nanosheets.

**Figure 3:**
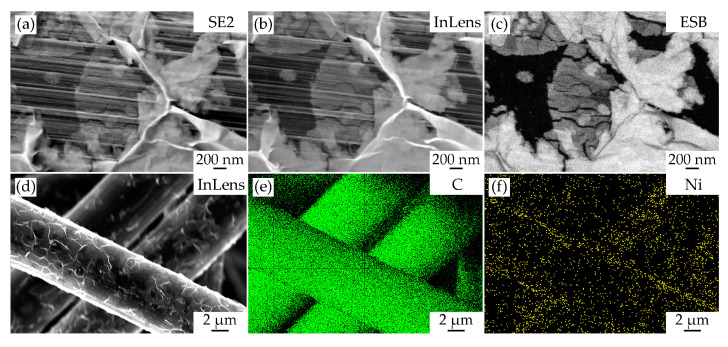
Microstructure of the contact area between the NiO nanosheets and the carbon fiber surface observed using different detectors (**a**–**c**), as well as maps of carbon (**e**) and nickel (**f**) distribution over the surface of the obtained NiO/CP electrode area (**d**).

The microstructure of the contact area between the NiO nanosheets and the carbon fiber surface was analyzed in more detail by scanning electron microscopy using different detectors (Figure 3a–c). As can be seen from the micrographs (Figure 3a) obtained with an Everhart–Thornley secondary electron detector (SE2) and well reflecting the material topography, some individual oxide nanosheets directly adhere to the carbon fiber surface, completely repeating its ribbed topography. At the same time, despite a rather low accelerating voltage (1 kV), these nanosheets appear translucent for the electron beam, which further confirms their thin-film nature.

The nickel oxide nanosheets’ structure can be observed more clearly as a result of the secondary electron detector (InLens), which allows us to form an image of the scanned surface with higher resolution (Figure 3b). In particular, individual nanosheets of small size in the lateral plane (about 200–300 nm in diameter), which consist of smaller planar particles (about 20–50 nm in diameter) and are probably the crystallization centers, which are formed at the initial stage of nanosheet enlargement, can be observed on some areas of the carbon fiber surface. The phase contrast mode image of the investigated area (Figure 3c) acquired with the backscattered electron detector (ESB) further confirms that the observed cellular and hierarchically organized nanosheet-based structures are related to nickel oxide and are on the surface of a carbon substrate characterized by a significantly lower molar mass value. Also, the image of the surface in the mode of average atomic number distribution according to the gray shade intensity enables us to distinguish areas within the oxide shell, which differ in thickness.

Simultaneously, with the study of the microstructural features of the obtained NiO/CP composite electrode, maps of carbon (Figure 3e) and nickel (Figure 3f) distribution over the surface of the studied material (Figure 3d) were also plotted with the use of energy-dispersive X-ray microanalysis. As can be seen from the corresponding micrograph, even though the formed oxide film consists of very thin nanosheets, it nevertheless shows up quite well using an in-lens secondary electron detector, even at a high electron beam accelerating voltage (20 kV). When examining the obtained element distribution maps, it can be seen that due to the thin-film nature of the oxide film and the sufficiently deep penetration of the electron beam, carbon is observed as the main chemical element on the surface of the fibers. At the same time, on the surface of carbon fibers, to a much lesser extent but evenly distributed, is nickel, which belongs to the NiO shell. The results of elemental analysis of the surface of individual fibers in this case allowed us to establish the following content of its constituent elements (at. %): C (96.6), O (3.3), Ni (0.1). Taking into account the fact that carbon and oxygen are light elements, the concentration of which this method allows us to determine with a sufficiently large error, the results obtained cannot be considered quantitative, but they confirm the presence of nickel oxide on the carbon fiber surface, as well as the thin-film structure of the hierarchically organized NiO shell formed in hydrothermal conditions.

**Figure 4:**
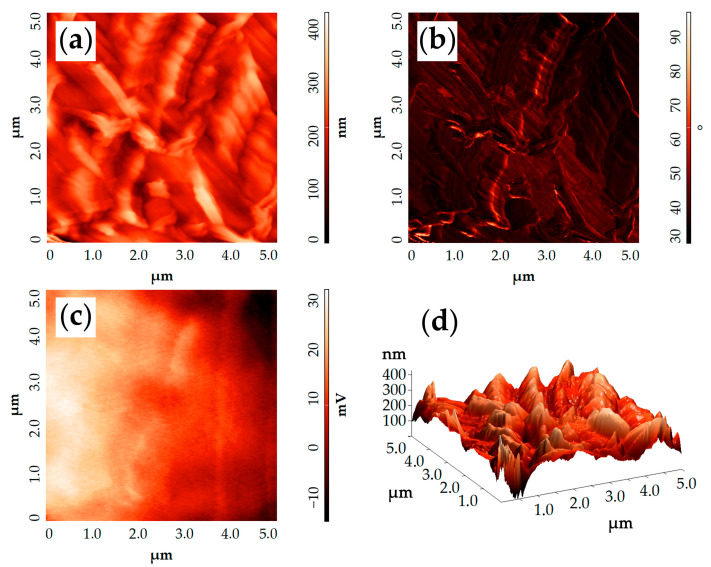
AFM results of NiO shell on carbon fiber: (**a**) topography, (**b**) relief in the phase contrast mode, (**c**) surface potential distribution map (according to KPFM data), (**d**) 3D image.

The AFM data on the study of a single carbon fiber surface coated with NiO film (Figure 4) are in good agreement with the SEM data. Thus, the topographic image (Figure 4a) shows complex-shaped NiO nanosheets, the edges and folds of which are oriented vertically (rising by 100–150 nm) relative to the carbon fiber surface. The resultant root mean square surface roughness of the coating was 43 nm. Furthermore, it can be seen that the oxide nanosheets tightly adhere to the carbon fiber surface and repeat its ribbed relief, which indicates their thin-film structure and high adhesion. The phase contrast image demonstrates (Figure 4b) that the probe was in the oscillation phase <90° almost all the time while scanning the studied area, which clearly indicates the absence of artifacts in the obtained topographic scan. Thus, with the help of AFM, the material’s morphology was examined sufficiently reliably and, for its additional visualization, a 3D image of the surface is given (Figure 4d).

The surface potential distribution map (Figure 4c) plotted in the course of KPFM measurements indicates that this parameter is distributed relatively uniformly over the studied area of the modified carbon fiber surface: the dispersion in values over an area of 25 μm^2^ reaches only ~45 mV, and the surface topography has little effect on potential distribution, although the maximum height difference in the topographic scan reaches 400 nm. The results indicate a high conductivity of the material, i.e., the nickel oxide film does not act as a significant barrier to charge transfer over the surface of the formed NiO/CP composite electrode. The work function value of the material surface calculated using the surface potential distribution map was 4.73 eV, which is slightly lower than the traditional values of this parameter for carbon fibers (4.95 eV) [53] or carbon nanoparticles (4.90–5.05 eV) [54,55], but very close to the work function values for nickel oxide obtained using the hydrothermal synthesis techniques that we use: 4.75 eV [56] and 4.70 eV [57]. In this connection, the results obtained by the AFM method demonstrate that a sufficiently homogeneous cellular film of nickel oxide was formed on almost the entire carbon fiber surface in hydrothermal conditions which, in some areas, tightly adheres to the carbon fiber surface and completely follows its topography.

**Figure 5:**
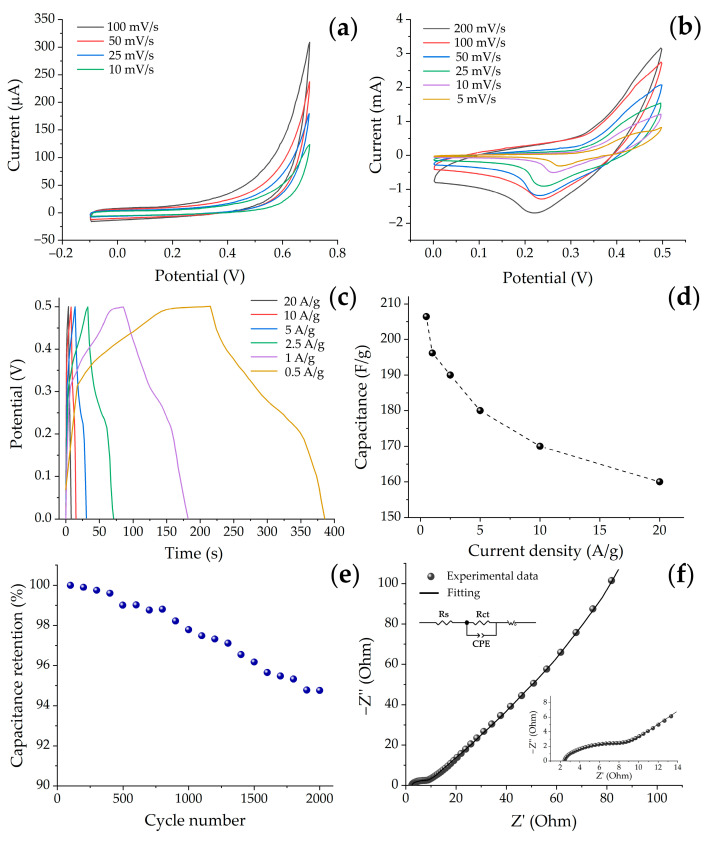
Cyclic voltammograms of pure CP (**a**) and NiO/CP electrode (**b**) in 3 M KOH solution obtained at different scan rates, GCD curves at different current densities (**c**), rate capability (**d**), cycling life test at 5 A/g for 2000 cycles (**e**), and the EIS Nyquist plot for NiO/CP electrode (**f**) as well as the enlargement of high-frequency part and equivalent circuit diagram ((**f**), inset).

Further, the electrochemical characteristics of the obtained NiO/CP electrode were investigated in a three-electrode scheme. As a comparison, Figure 5a,b shows cyclic voltammetry curves of the initial substrate, as well as the substrate with the grown film, registered at different scanning rates in 3M KOH medium. It can be seen that in the case of a clean substrate, the voltammetry patterns typical for non-activated carbon materials were obtained without any redox peaks [58]. At the same time, the response currents over the entire investigated potential range from −0.1 to 0.7 V are approximately an order of magnitude lower compared to the NiO/CP electrode, which indicates a negligibly small contribution of the substrate to the total electrode capacitance. Application of the active NiO layer (Figure 5b) to the carbon paper surface leads to a change in the CVA curve shape and the occurrence of two redox peaks, indicating the reversible transition between Ni^2+^ and Ni^3+^ taking place during Faraday reactions in the electrolyte medium, as well as the pseudocapacitance contribution to the total capacitance [59]:

NiO + OH^−^ ↔ NiOOH + e^−^ (1)

The redox processes taking place are caused mainly by the incorporation of OH groups from the electrolyte into the nickel oxide nanostructure during anodic and cathodic reactions, which further indicates the advantages of the porous or cellular microstructures of the active nickel oxide material in terms of increasing the amount of energy stored. It should also be noted that besides the change in the shape of the electrode CVA curve compared to similar curves for the substrate, there is also a significant increase in the area under the curves, which further points to an enhancement of the total electrode capacitance and a considerable contribution of the active layer [60].

In order to provide more detailed analysis of the formed NiO/CP electrode’s electrochemical behavior, galvanostatic charge–discharge tests were performed in the potential range of 0–0.5 V at current densities of 0.5–20 A/g. The specific capacitance (C_g_) values of the electrode material under study were calculated on the basis of the discharge curves data according to the equation [20]:

C_g_ = (I × Δt)/(m × ΔV), (2)

where I is the direct current value (A), Δt is the discharge time (s), and m is the mass of the active electrode material (g); ΔV is the potential window during the discharge.

The obtained curves (Figure 5c) are characterized by a non-linear change in the voltage magnitude over time during the charge–discharge process, which confirms the occurrence of Faraday processes when cycling the material. As the current density increases, the discharge time decreases; however, the curves’ shape is preserved without significant changes, indicating a high stability of the electrode material. The specific capacitance value of the NiO/CP electrode decreases from 207 to 160 F/g with increasing current density from 0.5 to 20 A/g (Figure 5d). The observed trend is characteristic of the electrode materials and is due to the diffuse limitations of the electrolyte ions’ access to the active sites in the material at high current densities [60]. The cycling life test during a process of 2000 charge/discharge cycles showed that the specific capacitance remains at 95% at a current density of 5 A/g (Figure 5e), which may be related to the features of the NiO film hierarchical structure.

Further, the obtained NiO/CP electrode was investigated by electrochemical impedance spectroscopy in the frequency range of 100 kHz–0.1 Hz. It can be seen (Figure 5f) that the resulting impedance spectrum consists of a semicircle and a linear part. The intersection point of the EIS curve with the real axis in the high-frequency range represents series resistance R_s_ (1.62 Ohm) and corresponds to the sum of the electrolyte resistance and the contact resistance occurring between the active material and the carbon paper substrate. The diameter of the semicircle observed in the hodograph in the middle-frequency range corresponds to the ion/electron charge transfer resistance R_ct_ (12.30 Ohm). Since the active material under study shows relatively low values of R_s_ (1.62 Ohm) and R_ct_ (12.30 Ohm), it can be assumed that in this case there is rather active ion transport at the electrode–electrolyte boundary, which can have a positive effect on the rate performance of the electrode. The linear component of the EIS spectrum can be described by the Warburg impedance (W_0_) corresponding to the diffusion of electrolyte ions to the active sites of the electrode material.

Thus, the capacitance values of the cellular NiO film grown on the carbon paper surface using the hydrothermal method generally correspond to the values for the electrode materials of nickel oxide-based supercapacitors [20,61,62,63]. An additional increase in the capacitance value could probably be achieved by increasing the concentration of the used reagents in the reaction system composition in order to form a thicker oxide film on the carbon fiber surface. It should be noted that this work demonstrates a promising one-pot hydrothermal synthesis of NiO as a thin hierarchically organized film on the surface of a flexible carbon paper substrate. The resulting material demonstrated high cycling stability (95% capacitance retention during 2000 cycles at 5 A/g), as well as specific capacitance values (207 F/g at 0.5 A/g), allowing us to consider it as a promising component of environmentally friendly flexible μ-supercapacitors.

## 4. Conclusions

This study investigated the formation of a cellular hierarchically organized NiO film on a carbon paper substrate under hydrothermal conditions as a flexible composite NiO/CP supercapacitor electrode. Using TGA/DSC analysis, the thermal behavior of the carbon paper substrate with the semi-product coating was studied and it was shown that such modification of the material surface leads to a noticeable increase in its thermal stability. Using SEM, it was shown that the NiO film grown on the carbon fiber surface under hydrothermal conditions is characterized by a complex cellular morphology, made of partially overlapping individual nanosheets of about 4–5 nm thickness and lateral dimensions up to 1–2 μm, some edges and folds of which are located vertically relative to the surface of the carbon fiber. When studying the contact area between the NiO nanosheets and the carbon fiber surface, it was found that some individual oxide nanosheets directly adhere to its surface, completely repeating its ribbed topography. It was shown that some parts of the carbon fiber surface contain individual nanosheets of small size in the lateral plane (about 200–300 nm in diameter), which consist of smaller flat particles (about 20–50 nm in diameter) and are probably the centers of crystallization, being formed at the initial stage of nanosheet enlargement. The obtained results of energy-dispersive X-ray microanalysis confirm the presence of nickel oxide on the carbon fiber surface, as well as the thin film structure of the hierarchically organized NiO shell formed in hydrothermal conditions. The results of AFM are in good agreement with the SEM data and confirm the cellular structure of the oxide film, as well as the presence of thin oxide nanosheets on the carbon fiber surface tightly adhering to them and repeating their topography. The electronic work function of the oxide film surface was estimated by KPFM with a value of 4.73 eV. The electrochemical characteristics of the prepared flexible NiO/CP electrode were investigated in the framework of the three-electrode scheme. As a result, the obtained material showed high cycling stability (95% capacitance retention during 2000 cycles at 5 A/g) and values of specific capacitance (207 F/g at 0.5 A/g), allowing us to consider it as a promising component of environmentally friendly flexible μ-supercapacitors.

## Data Availability

Not applicable.
